# Seeing Angiogenesis: Multiparametric Ultrasound as a Non-Invasive Window into the Vascularization Status of Cervical Lymph Nodes in Head and Neck Squamous Cell Carcinoma

**DOI:** 10.3390/medicina62091684

**Published:** 2026-09-02

**Authors:** Marius Liviu Moise, Cristina Stefania Dumitru, Dorin Novacescu, Alina Cristina Barb, Diana Szekely, Alexia Manole, Cristian Silviu Suciu, Flavia Zara, Raul Patrascu

**Affiliations:** 1 Radiology Department, Emerald Medical Center, Corneliu Coposu Boulevard 14, 300552 Timisoara, Romania; moiseliviumarius@gmail.com; 2 Department II of Microscopic Morphology, Discipline of Histology, “Victor Babes” University of Medicine and Pharmacy Timisoara, E. Murgu Square, No. 2, 300041 Timisoara, Romania; novacescu.dorin@umft.ro (D.N.); toma.alina@umft.ro (A.C.B.); cristian_suciu@umft.ro (C.S.S.); flavia.zara@umft.ro (F.Z.); 3 Doctoral School, “Victor Babes” University of Medicine and Pharmacy Timisoara, E. Murgu Square, No. 2, 300041 Timisoara, Romania; 4 Faculty of Medicine and Pharmacy, University of Oradea, 410087 Oradea, Romania; manole.alexia@student.uoradea.ro; 5 Department of Functional Sciences, “Victor Babes” University of Medicine and Pharmacy, 300041 Timisoara, Romania; patrascu.raul@umft.ro

**Keywords:** head and neck squamous cell carcinoma, ultrasound, angiogenesis, contrast-enhanced ultra-sound, elastography, vascular endothelial growth factor, cervical lymph node, imaging biomarker

## Abstract

Cervical lymph node status is the single most important prognostic determinant in head and neck squamous cell carcinoma (HNSCC), yet conventional gray-scale ultrasound relies on morphological criteria that only indirectly reflect the underlying biological process driving nodal progression: tumor angiogenesis. This narrative review focuses specifically on cervical lymph nodes and examines whether multiparametric ultrasound (B-mode, Doppler, elastography, and contrast-enhanced ultrasound [CEUS]) can serve as a non-invasive, repeatable surrogate for nodal angiogenic activity in HNSCC and outlines a practical framework for its use in the head and neck clinic; evidence on the primary tumor itself was outside this review’s scope. A narrative synthesis of the literature on angiogenesis biology in HNSCC and on multiparametric ultrasound of cervical lymph nodes was performed, drawing on systematic reviews, meta-analyses, and clinical pilot studies identified through PubMed-indexed sources. Vascular endothelial growth factor (VEGF)-driven neoangiogenesis is a recognized diagnostic, prognostic, and predictive biological axis in HNSCC. Ultrasound modalities that visualize or quantify microvascular architecture and perfusion—Doppler vascularity patterns, shear-wave elastography, and CEUS—track features that are biologically continuous with angiogenesis (disorganized, high-permeability neovessels; altered tissue stiffness from stromal remodeling). Combining CEUS with B-mode ultrasound improves diagnostic accuracy for inconclusive cervical lymph nodes compared with either modality alone, and pilot multiparametric protocols in HNSCC show reproducible irregular contrast enhancement and elastographic heterogeneity in metastatic nodes. Multiparametric ultrasound does not replace histology, but it offers a repeatable, radiation-free, low-cost window onto the vascular phenotype of HNSCC that complements morphological staging. Given the heterogeneity and methodological limitations of the available evidence, multiparametric ultrasound should currently be regarded as a promising imaging biomarker rather than an established non-invasive surrogate for angiogenesis. Larger prospective, HNSCC-specific cohorts using standardized acquisition protocols, with direct histological correlation, are needed before this approach can be adopted into routine diagnostic pathways.

## 1. Introduction

Head and neck squamous cell carcinoma (HNSCC) is a heterogeneous group of malignancies arising from the mucosal epithelium of the oral cavity, pharynx, larynx, and related anatomical sites. Together with cancers of the lip and salivary glands, HNSCC represents the seventh most common cancer worldwide, accounting for approximately 890,000 new cases and 450,000 deaths annually according to GLOBOCAN estimates [1,2,3,4]. Despite continuous advances in surgery, radiotherapy, systemic therapy, and immunotherapy, prognosis remains strongly dependent on disease stage at diagnosis. Among all prognostic variables, cervical lymph node metastasis is the single most important determinant of survival, reducing disease-specific survival by nearly 50% regardless of the primary tumor site and significantly increasing the risk of locoregional recurrence and distant metastasis [2,5,6].

The diagnostic evaluation of cervical lymph nodes in patients with suspected HNSCC relies on clinical examination combined with cross-sectional imaging, including computed tomography (CT), magnetic resonance imaging (MRI), positron emission tomography/computed tomography (PET/CT), and ultrasound (US). Owing to its wide availability, low cost, absence of ionizing radiation, and excellent spatial resolution for superficial structures, ultrasound remains the first-line imaging modality for the assessment of cervical lymphadenopathy in routine clinical practice [7,8,9]. Conventional grayscale (B-mode) ultrasound identifies suspicious lymph nodes using morphological criteria such as a rounded shape, increased short-axis diameter, absence of the echogenic fatty hilum, cortical heterogeneity, cystic degeneration, microcalcifications, and irregular nodal margins. Although these features provide valuable diagnostic information, they mainly represent structural consequences of malignant infiltration rather than the biological mechanisms driving tumor progression [7,8,9,10].

One of the fundamental biological processes underlying HNSCC progression is angiogenesis. Tumor angiogenesis is characterized by the formation of structurally abnormal, tortuous, and highly permeable blood vessels that support tumor growth, invasion, and metastatic dissemination. This process is orchestrated predominantly through vascular endothelial growth factor (VEGF) signaling, together with hypoxia-inducible factor-1α (HIF-1α), fibroblast growth factors, platelet-derived growth factor, transforming growth factor-β, inflammatory cytokines, and interactions within the tumor microenvironment [2,11,12,13,14]. Numerous studies have demonstrated that increased microvascular density and elevated VEGF expression correlate with advanced tumor stage, cervical lymph node metastasis, treatment resistance, and poor overall survival in patients with HNSCC, establishing angiogenesis as an important diagnostic, prognostic, and predictive biomarker [11,12,13,14,15].

Importantly, angiogenesis produces structural and functional alterations that can be detected by advanced ultrasound techniques. Neovascularization results in abnormal vascular architecture, heterogeneous tissue perfusion, increased vascular permeability, extracellular matrix remodeling, fibrosis, and changes in tissue stiffness, all of which have measurable imaging correlates [11,16,17]. Consequently, ultrasound techniques capable of evaluating vascularity and tissue biomechanics may provide indirect but clinically relevant information regarding tumor angiogenic activity.

Color and power Doppler ultrasound evaluate macroscopic vascular distribution and intranodal blood flow patterns, providing information on vascular density and vessel organization. Shear-wave elastography quantitatively measures tissue stiffness, a non-specific parameter reflecting extracellular matrix remodeling, fibrosis, desmoplasia, tumor cellularity, and, in previously treated patients, post-radiotherapy change—of which angiogenesis-associated neovascularization is only one contributor rather than the dominant or distinguishing one. Contrast-enhanced ultrasound (CEUS), using purely intravascular microbubble contrast agents, enables real-time visualization of microvascular perfusion at the capillary level, generating both qualitative enhancement patterns and quantitative time–intensity curve parameters that reflect tissue perfusion dynamics [17,18,19,20,21]. Individually, each modality offers complementary information; collectively, they constitute the concept of multiparametric ultrasound, shifting sonographic evaluation from purely morphological assessment toward functional characterization of the tumor vascular phenotype.

This review has two principal objectives. First, it summarizes the biological basis linking angiogenesis to tumor progression, metastatic behavior, and prognosis in HNSCC. Second, it critically reviews the current evidence regarding Doppler ultrasound, elastography, and contrast-enhanced ultrasound in the evaluation of cervical lymph nodes, discussing the strengths, limitations, and future potential of multiparametric ultrasound as a non-invasive imaging biomarker of angiogenesis in patients with head and neck squamous cell carcinoma.

Within the current head and neck cancer diagnostic pathway, multiparametric ultrasound is not proposed as a replacement for CT, MRI, or PET/CT but as a complementary, radiation-free modality particularly suited to two roles: cervical nodal assessment and image-guided tissue sampling. Rather than presenting it broadly as an angiogenesis tool, this review specifically identifies three clinical scenarios in which it is positioned to add value alongside cross-sectional imaging: (i) further characterization of cervical lymph nodes that remain equivocal after B-mode assessment or CT/MRI/PET-CT, (ii) guidance of targeted biopsy toward the region of a node most likely to be angiogenically active, and (iii) surveillance of the post-radiotherapy neck, where fibrosis and architectural distortion limit the reliability of morphological criteria alone. These scenarios, detailed in Section 5, frame the evidence reviewed below.

## 2. Methodology

This is a narrative review; no formal systematic-review protocol was registered, and no PRISMA-style study-flow diagram or risk-of-bias scoring was applied. The aim was to synthesize the biological rationale for angiogenesis as a driver of HNSCC behavior and to map it against the available evidence on multiparametric ultrasound (B-mode, Doppler, elastography, and contrast-enhanced ultrasound) of cervical lymph nodes, rather than to exhaustively quantify effect sizes across the entire literature.

Literature was identified through a PubMed search performed on 15 July 2026, restricted to the period January 2016–July 2026, English language, and human studies, using the following search string: (“head and neck squamous cell carcinoma”[tiab] OR “head and neck cancer”[tiab] OR “HNSCC”[tiab]) AND (“cervical lymph node”[tiab] OR “cervical lymph nodes”[tiab] OR “neck lymph node*”[tiab]) AND (“ultrasound”[tiab] OR “ultrasonography”[tiab] OR “Doppler”[tiab] OR “elastography”[tiab] OR “shear wave”[tiab] OR “contrast-enhanced ultrasound”[tiab] OR “CEUS”[tiab]) AND (“angiogenesis”[tiab] OR “vascular endothelial growth factor”[tiab] OR “VEGF”[tiab] OR “microvessel density”[tiab]). Titles and abstracts returned by this search were screened independently by two authors against the inclusion criteria stated below; full texts were retrieved for any record either screener flagged as potentially eligible. PubMed was selected as the single search engine because it indexes MEDLINE together with the great majority of peer-reviewed head and neck oncology and radiology journals relevant to this topic and because it was judged sufficient for a narrative, rather than systematic, synthesis; we acknowledge in the Limitations that this single-database approach may have missed literature indexed only in Embase, Scopus, or Web of Science. The lower boundary of 2016 was chosen because it corresponds to the publication of the EFSUMB guidelines on the clinical use of contrast-enhanced ultrasound in non-hepatic applications [17], after which the technical parameters and reporting terminology for Doppler, elastography, and CEUS in superficial organs became sufficiently standardized to allow meaningful comparison across studies; earlier reports, particularly on CEUS, used heterogeneous, pre-standardization protocols that are difficult to interpret alongside contemporary evidence. This boundary applies to the ultrasound-technique evidence base specifically; it was not intended to, and does not, restrict the biological foundations of this review. Foundational concepts in tumor angiogenesis and VEGF signaling substantially predate 2016, and this review draws on them throughout Section 3 via subsequent authoritative reviews and syntheses that themselves incorporate the seminal original work, rather than by re-citing primary discovery-era papers directly. Reference lists of retrieved systematic reviews and meta-analyses were screened for additional relevant primary studies, partially mitigating the single-database restriction. Priority was given, where available, to systematic reviews and meta-analyses over individual primary studies. In practice, this meant that when a systematic review or meta-analysis addressing a given technique (e.g., SWE for cervical lymph node discrimination) was identified, it was cited as the primary source for pooled diagnostic accuracy, and individual primary studies already incorporated into that synthesis were not separately re-cited; primary studies were cited in their own right only when they addressed a question not covered by any available systematic review or meta-analysis, such as a technique applied specifically to HNSCC, a recent study published after the most current synthesis, or a methodological point (e.g., comparative imaging economics, biopsy technique) outside the scope of the diagnostic-accuracy syntheses. This approach was adopted because pooled, systematically synthesized estimates are less susceptible to the selection and reporting biases of any single primary study and therefore offer a more stable summary of diagnostic performance, even though it does not eliminate the heterogeneity of the underlying primary literature, which is addressed explicitly in Section 6 (Limitations). Because the HNSCC-specific multiparametric ultrasound literature remains limited, representative studies from papillary thyroid carcinoma—a histologically distinct but sonographically well-characterized model of cervical nodal metastasis—were included where they illustrate a technique or finding not yet independently replicated in HNSCC; this substitution is flagged explicitly at each point in the text where it occurs, and its implications for extrapolation are addressed in the Discussion. Studies were considered for inclusion if they reported original human data or a systematic synthesis thereof, addressed cervical lymph node characterization in the context of head and neck or thyroid malignancy, and were published in English in a peer-reviewed journal. The English language restriction was adopted for feasibility of full-text screening and data extraction by the review team; we cannot exclude the possibility that relevant non-English-language literature, particularly from high-incidence regions for HNSCC, was missed as a result, and we note this explicitly as a limitation in Section 6. Conference abstracts, case reports, and non-peer-reviewed sources were excluded. No minimum sample size was imposed, given the acknowledged scarcity of HNSCC-specific multiparametric ultrasound cohorts; where a cited primary study is a small pilot series, this is stated explicitly alongside the finding.

## 3. Angiogenesis in HNSCC: The Biological Rationale

Angiogenesis—the sprouting of new blood vessels from pre-existing vasculature—is recognized as one of the core hallmarks of malignant transformation, enabling tumors to overcome the diffusion limit of oxygen and nutrients (approximately 100–200 µm) that would otherwise constrain solid tumor growth to a few cubic millimeters [13]. As a tumor mass expands beyond this limit, hypoxic cores develop, triggering a cascade of adaptive signaling that shifts the local balance of pro- and anti-angiogenic factors toward vessel formation—a transition often termed the “angiogenic switch” [10].

The central effector of this switch is vascular endothelial growth factor (VEGF), a family of secreted glycoproteins (VEGF-A, -B, -C, -D, and placental growth factor) acting through receptor tyrosine kinases VEGFR-1, -2, and -3 expressed on vascular and lymphatic endothelium. VEGF-A/VEGFR-2 signaling is the principal driver of pathological neoangiogenesis, promoting endothelial cell proliferation, migration, and increased vascular permeability, while VEGF-C/VEGFR-3 signaling additionally supports lymphangiogenesis and, by extension, lymphatic spread [10,14]. In HNSCC specifically, VEGF expression has been repeatedly associated with advanced clinical stage, cervical lymph node involvement, and reduced overall survival and is increasingly regarded as a diagnostic, prognostic, and predictive biomarker across oral, laryngeal, and pharyngeal subsites [10].

Upstream of VEGF, tumor hypoxia acts as a principal trigger through stabilization of hypoxia-inducible factor-1α (HIF-1α), a transcription factor that, under low-oxygen conditions, escapes proteasomal degradation and induces transcription of VEGF and a broader hypoxia-response gene program governing glycolytic metabolism, cell survival, and invasion. In oral squamous cell carcinoma, a meta-analysis of 18 studies (1474 patients) found HIF-1α overexpression significantly associated with larger tumor size, advanced TNM stage, and reduced overall survival, supporting its role as an independent prognostic marker upstream of the angiogenic cascade [22].

At the tissue level, this biology is most directly captured by microvessel density (MVD), quantified histologically using endothelial markers such as CD31 and CD105/endoglin. Because CD105 is preferentially expressed on actively proliferating, tumor-associated endothelium rather than on quiescent pre-existing vessels, it is considered a more specific surrogate of ongoing neoangiogenesis than pan-endothelial markers, and higher CD105-assessed MVD has been linked to loco-regional recurrence and reduced disease-free survival in HNSCC across multiple series [10].

Direct evidence from HNSCC tissue supports this framework: in a cohort analysis correlating VEGF immunohistochemical expression with clinical TNM staging, VEGF expression increased progressively with tumor stage and was associated with markers of tumor progression, reinforcing VEGF as a tractable biological readout of disease aggressiveness rather than a purely mechanistic curiosity [11].

Beyond its direct vascular effects, VEGF signaling also shapes the tumor immune microenvironment. VEGF suppresses dendritic cell maturation, impairs cytotoxic T-lymphocyte infiltration, and has been shown in HNSCC to correlate with markers of local immunosuppression, providing a mechanistic link between angiogenic activity and the immune-evasive phenotype associated with treatment resistance [14].

**Figure 1:**
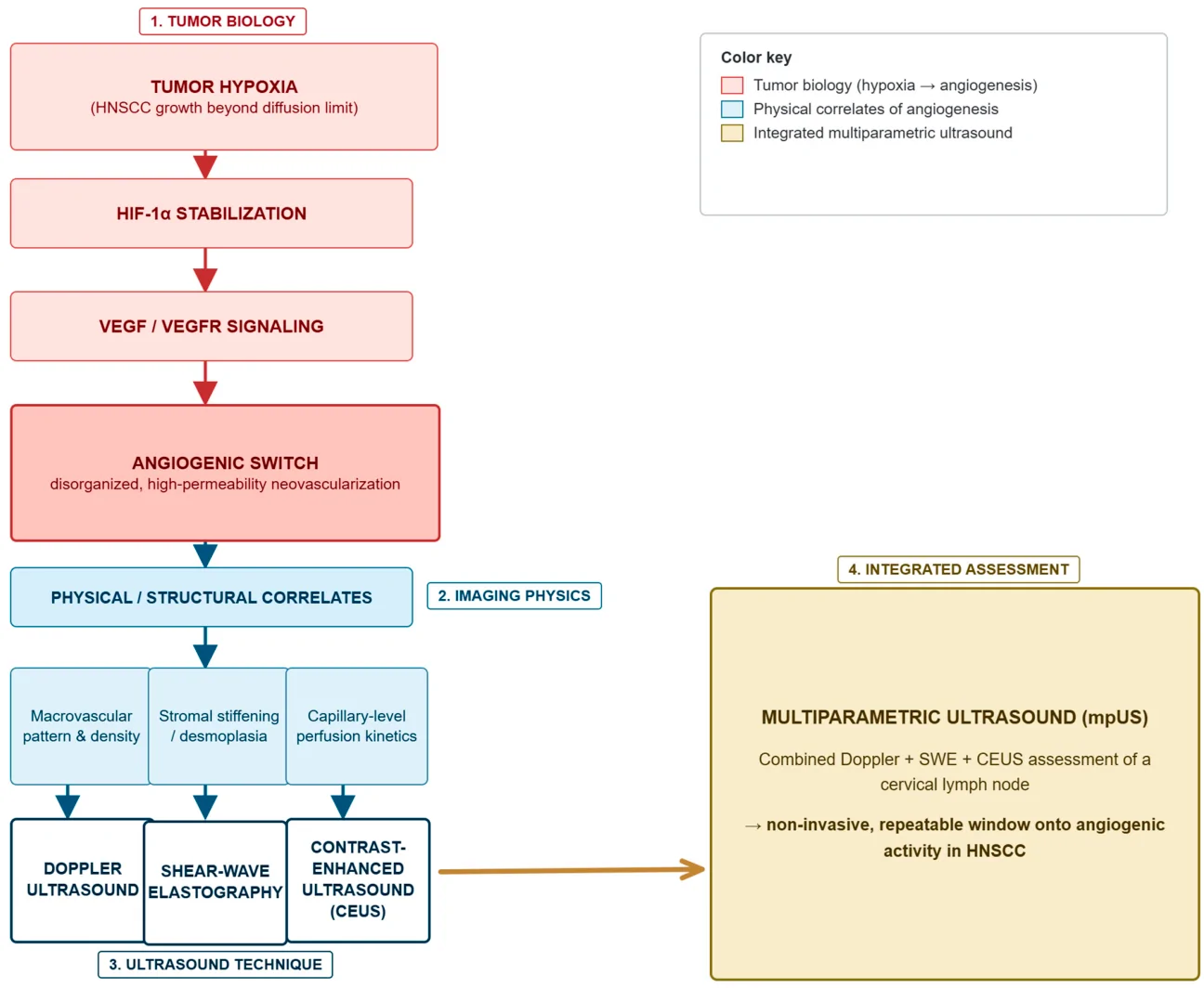
Proposed biological-to-imaging pathway linking hypoxia-driven angiogenesis in head and neck squamous cell carcinoma (HNSCC) to multiparametric ultrasound findings. Tumor hypoxia stabilizes HIF-1α, which upregulates VEGF/VEGFR signaling and triggers the angiogenic switch. The resulting disorganized, high-permeability neovascularization produces three physical correlates—macrovascular pattern, stromal stiffening, and capillary-level perfusion—detectable respectively by Doppler ultrasound, shear-wave elastography (SWE), and contrast-enhanced ultrasound (CEUS). Combined (multiparametric) assessment of these three modalities is proposed as a non-invasive, repeatable window onto angiogenic activity in HNSCC cervical lymph nodes.

This biology has direct therapeutic relevance: anti-angiogenic agents targeting VEGF or VEGFR (e.g., bevacizumab, multikinase inhibitors) have been evaluated in HNSCC on the rationale that disrupting this axis should limit tumor growth and improve the efficacy of concurrent chemoradiation. Clinical benefit has, however, been modest and inconsistent, in part because compensatory pro-angiogenic pathways (FGF, PDGF, angiopoietins) can sustain vascularization when VEGF signaling alone is blocked [12]. This therapeutic ambiguity is itself a further argument for better non-invasive characterization of angiogenic activity in individual tumors—a diagnostic gap that multiparametric ultrasound is positioned to address, as summarized in Figure 1 and discussed in detail in the following section.

## 4. Ultrasound Windows onto the Angiogenic Phenotype

**Figure 2:**
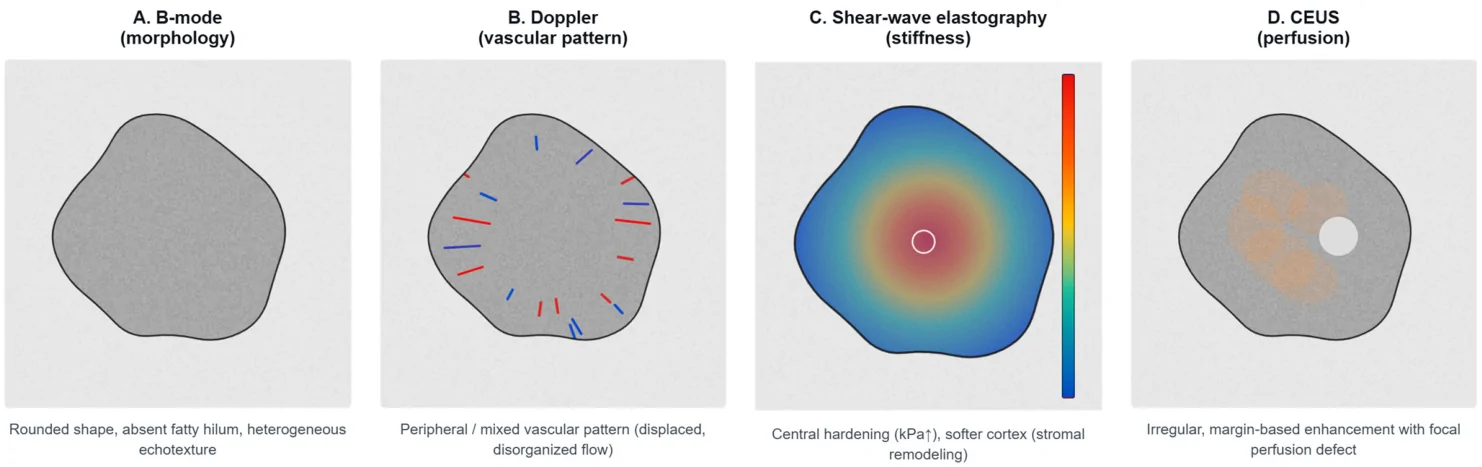
Schematic representation of a metastatic cervical lymph node as expected to appear across the ultrasound modalities discussed in this review. (**A**) B-mode: rounded shape, absent fatty hilum, and heterogeneous echotexture. (**B**) Doppler: peripheral or mixed vascular pattern, reflecting displaced, disorganized neovascular flow rather than the normal central hilar vessel. (**C**) Shear-wave elastography: a stiffer central core surrounded by a relatively softer cortex, consistent with centrally dense angiogenic and desmoplastic stromal remodeling; color scale indicates relative stiffness (blue = soft, red = stiff). (**D**) Contrast-enhanced ultrasound (CEUS): irregular, margin-based enhancement with a focal perfusion defect corresponding to necrosis. This is an illustrative schematic synthesizing the qualitative findings described in Section 4 and is not a reproduction of an actual patient ultrasound examination.

The biological cascade outlined above—hypoxia, HIF-1α stabilization, VEGF-driven neovascularization, and the resulting disorganized, high-permeability vasculature—is not a purely histological abstraction. Each step leaves a physical signature that can, at least in principle, be captured by an ultrasound machine: macroscopic vascular pattern by Doppler, stromal stiffening by elastography, and capillary-level perfusion kinetics by contrast-enhanced ultrasound (CEUS). Figure 2 summarizes, schematically, how a metastatic cervical lymph node is expected to appear across these four modalities; the following subsections review the empirical evidence supporting each panel individually.

### 4.1. B-Mode and Doppler Ultrasound

Conventional gray-scale (B-mode) ultrasound remains the mandatory first step in any cervical node assessment, and its criteria—rounded shape, absence of the echogenic fatty hilum, cortical heterogeneity, cystic change, and microcalcifications—are well established in the head and neck imaging literature [7]. The scale at which these criteria have now been validated is considerable: in a prospective cohort of 235 patients with HNSCC undergoing meticulous node-by-node correlation between preoperative ultrasound and histopathology, 4539 lymph nodes were assessed sonographically and 7237 examined histologically, with 2684 nodes evaluated by both methods. Subjective B-mode assessment by an experienced examiner achieved a sensitivity of 79% and specificity of 99% for metastatic involvement, and optimal cut-off points for nodal roundness indices were derived separately for each AAO-HNS neck level, reflecting the fact that normal nodal morphology varies systematically by anatomical location [8]. Notably, predictive performance improved with increasing size of the metastatic deposit, and the smallest short-axis diameter combined with subjective B-mode impression gave the best overall agreement with pathological N-stage (Cohen’s κ = 0.713)—underscoring that morphology alone still struggles most with the sub-centimeter, early-stage deposits that matter most for treatment planning [8].

Doppler ultrasound adds a first layer of functional, vascularity-based information beyond static morphology. Benign and reactive nodes typically show a preserved hilar or central vascular pattern, whereas malignant infiltration displaces, compresses, or destroys the normal hilar architecture, producing peripheral, mixed, or diffusely scattered flow signals—a pattern directly attributable to the disorganized angiogenic vasculature described in Section 3. In a prospective study of 102 HNSCC patients (211 nodes) undergoing ultrasound-guided fine-needle aspiration cytology, the addition of micro-flow imaging (MFI)—a sensitive power-Doppler-based technique for depicting microvascular flow—together with resistive index measurement and assessment of the fatty hilum sign meaningfully improved the pre-test selection of nodes worth aspirating, with strong peripheral vascularity on MFI serving as a specific indicator of malignancy even in nodes that were otherwise morphologically ambiguous [23]. This is a direct, clinically actionable illustration of the principle underlying this review: a vascular-pattern parameter, grounded in angiogenesis biology, adding diagnostic value beyond morphology alone.

A recent methodological development is worth noting here, as it illustrates where Doppler-based nodal assessment is heading. Eida et al. trained a YOLOv7-based deep learning model on 462 paired B-mode and power-Doppler (“D-mode”) ultrasound images from 126 HNSCC patients (261 metastatic and 279 non-metastatic histopathologically confirmed nodes) and found that the D-mode (Doppler) model’s detection performance approached that of highly experienced radiologists and exceeded that of less experienced residents, suggesting that vascular-pattern information is not only diagnostically informative but also learnable by automated systems, a point taken up further in Section 4.4 [24].

### 4.2. Shear-Wave Elastography

Shear-wave elastography (SWE) quantifies tissue stiffness by measuring the propagation velocity of acoustic radiation force-induced shear waves through tissue, expressed in kilopascals (kPa) or meters per second, and is formally standardized for non-hepatic applications—including superficial lymph nodes—in the current EFSUMB guideline [15]. Because SWE is automated rather than operator-compressed, it is inherently more reproducible than the older, semi-quantitative strain elastography it has largely superseded [19]. Biologically, the technique is relevant here because the desmoplastic, densely cellular stroma that accompanies tumor infiltration measurably stiffens tissue relative to normal or reactively hyperplastic nodal parenchyma. This stiffening, however, is not specific to angiogenesis: from a surgical and histopathological standpoint, tissue stiffness on SWE reflects a composite of fibrosis, desmoplasia, tumor cellularity, necrosis, and—in previously irradiated patients—post-radiotherapy fibrotic change, any of which can elevate stiffness independent of neovascularization. SWE should therefore be regarded as a marker of stromal remodeling broadly, not a specific surrogate for angiogenic activity; where this review refers to elastographic findings as “angiogenically active,” this denotes co-occurrence with angiogenesis within the malignant stroma, not a claim that stiffness itself is mechanistically specific to neovascularization.

The quantitative evidence base for this is substantial. A systematic review and meta-analysis pooling eight studies (481 patients, 647 cervical lymph nodes) reported a summary sensitivity of 81% and specificity of 85% for SWE in distinguishing malignant from benign cervical lymph nodes, with no significant difference in performance between the two principal SWE techniques (point-based acoustic radiation force impulse imaging and 2D supersonic shear imaging) [25]. A more recent, prospective single-center Romanian study (Cluj-Napoca) directly measured this stiffness gradient across pathology types: the maximum SWE value recorded among 39 benign lymph nodes was 35 kPa, compared to a minimum of 40 kPa among 14 lymphomatous nodes and a minimum of 50 kPa among 27 metastatic nodes (mean 122.8 kPa, substantially exceeding both other groups)—a clear, graded relationship between tissue stiffness and malignant pathology, consistent with a general pattern of stromal remodeling rather than a specific angiogenic signal. In multivariable analysis combining SWE value, patient sex, and nodal long-axis diameter, the final model discriminated metastatic from lymphomatous adenopathy with 88.9% sensitivity and 78.6% specificity (AUC 0.837), using an optimal SWE cut-off of 71.5 kPa [16]. While this cohort was not restricted to HNSCC (it also included lymphoma and other primary sites), the majority of its metastatic cases originated from oral squamous cell carcinoma, and the graded stiffness pattern it demonstrates—benign softest, lymphoma intermediate, carcinoma metastasis stiffest—is consistent with the broader stromal remodeling framework proposed in this review, though this study did not isolate angiogenesis from the fibrotic, desmoplastic, and cellularity-related contributors to stiffness discussed above, and its design cannot attribute the observed gradient specifically to neovascularization.

In HNSCC specifically, the pilot multiparametric protocol of Künzel et al. found irregular, clearly demarcated elastographic hardening localized to the center of every examined metastatic node (13/13 patients), with a significantly softer cortex relative to both the nodal center and surrounding tissue—an architectural pattern consistent with a densely remodeled malignant core surrounded by a less-affected periphery; however, as above, elastographic hardening alone cannot distinguish angiogenic stromal remodeling from fibrosis, desmoplasia, or necrosis within that core [20]. Taken together, these findings position SWE as a reproducible, quantitative complement to B-mode and Doppler assessment, capturing a biologically distinct dimension, stromal stiffness, that neither morphology nor vascular pattern alone can provide; it should be interpreted as a non-specific marker of stromal remodeling that co-occurs with angiogenesis in malignant tissue, not as a specific angiogenic biomarker in its own right.

### 4.3. Contrast-Enhanced Ultrasound (CEUS)

Of the three functional modalities considered in this review, CEUS is conceptually the most directly coupled to angiogenesis because it visualizes capillary-level microvascular perfusion in real time using intravenously administered, purely intravascular microbubble contrast agents, rather than inferring vascularity indirectly from Doppler shift or tissue stiffness. Both qualitative enhancement patterns (homogeneity, margin characteristics, presence of perfusion defects) and quantitative time–intensity curve (TIC) parameters (time-to-peak, area under the curve, wash-in and wash-out kinetics) can be extracted from a single CEUS examination, and its clinical use is now formally standardized in the current EFSUMB non-hepatic applications guideline [17], with detailed procedural guidance available in companion technical reviews [18]. The safety profile of the technique is notable: large retrospective series report adverse-event rates for ultrasound microbubble contrast agents on the order of 0.01–0.02%, and—unlike iodinated CT contrast or gadolinium-based MRI agents—the agents are eliminated pulmonarily rather than renally, permitting use in patients with renal impairment [26].

Malignant cervical lymph nodes typically display inhomogeneous, often centripetal or irregular-margin enhancement with focal perfusion defects corresponding to necrosis or chaotic flow, in contrast to the more homogeneous, hilum-centered enhancement of reactive nodes. In papillary thyroid carcinoma—the best-studied sonographic model of cervical nodal metastasis by sheer volume of literature—combining CEUS with elastography and conventional ultrasound in a logistic regression model improved discrimination between metastatic and reactive nodes beyond what any single modality achieved alone [9]. At the level of pooled quantitative evidence, a systematic review and meta-analysis of five eligible studies found that adding CEUS to conventional ultrasound raised pooled sensitivity from 76% (95% CI 66–83%) to 92% (95% CI 89–95%) and specificity from 80% (95% CI 45–95%) to 91% (95% CI 87–94%) for characterizing inconclusive cervical lymph nodes, with the combined modality also showing markedly lower between-study heterogeneity (I^2^ = 0%) than either technique alone, a meaningful statistical signal that the combination is not just more accurate but more consistently so [26].

In the HNSCC-specific pilot cohort of Künzel et al., early arterial-phase CEUS showed irregular, margin-based enhancement in nearly all examined metastatic nodes, together with a short time-to-peak (rapid wash-in) and high area-under-the-curve on TIC analysis relative to surrounding tissue—a perfusion signature entirely consistent with the high-flow, structurally leaky neovasculature that angiogenesis biology predicts [20]. This convergence between the histological picture described in Section 3 and the sonographic picture described here is, in essence, the central argument of this review.

### 4.4. Toward a Multiparametric Protocol

No single ultrasound technique captures the full angiogenic phenotype: Doppler depicts macrovascular architecture, SWE depicts the stromal consequence of angiogenic remodeling, and CEUS depicts capillary-level perfusion directly. The logical next step—and the one most directly relevant to this review’s central argument—is their combined, structured use within a single examination, an approach generally termed multiparametric ultrasound (mpUS).

The strongest currently available head-to-head evidence for this comes from Wakonig et al., who examined 107 inconclusive cervical lymph nodes in 105 patients using a complete mpUS protocol (B-mode, color-coded duplex sonography, SWE, and CEUS), evaluated in consensus against histopathological reference standard. The combination of SWE and CEUS achieved the highest overall diagnostic performance of any modality or pairing tested: 91% sensitivity, 77% specificity, 87% positive predictive value, and 83% negative predictive value [21]. A companion analysis by the same Berlin group, focused specifically on the quantitative (rather than purely qualitative) parameters obtainable from the same mpUS protocol, further supports the feasibility of extracting objective, numerically reproducible vascular and stiffness metrics from a single structured examination rather than relying solely on subjective pattern recognition [27].

The HNSCC-specific pilot protocol of Künzel et al.—though limited to 13 patients—remains the clearest existing demonstration that a full mpUS examination (B-mode, SWE, and CEUS performed sequentially in the same session) is feasible in routine pre-therapeutic HNSCC practice, and its findings are internally consistent: the same nodal regions showing elastographic hardening also showed irregular CEUS enhancement, suggesting that both parameters are tracking the same underlying angiogenically active tumor core rather than independent, unrelated phenomena [20]. This spatial concordance between stiffness and perfusion abnormality is, arguably, the strongest indirect evidence available that multiparametric ultrasound is measuring a coherent biological process—angiogenesis—rather than two unrelated imaging artifacts.

Finally, the emergence of deep learning-assisted interpretation, illustrated by the YOLOv7 model of Eida et al. applied to B-mode and Doppler images [24], points toward a plausible future direction for mpUS: automated, quantitative, less operator-dependent extraction of vascular and stiffness parameters across all three functional modalities simultaneously, potentially addressing the single greatest practical limitation of ultrasound-based angiogenesis assessment—its dependence on examiner experience. Whether such tools can be extended from single-modality (B-mode/Doppler) detection to genuinely multiparametric, quantitative angiogenic phenotyping remains an open and, at present, HNSCC-specific research question—one that connects directly to the clinical applicability considerations discussed in the following section.

The evidence reviewed across Section 4.1, Section 4.2, Section 4.3 and Section 4.4 is summarized in Table 1, which brings together study design, modality, and principal diagnostic findings across the HNSCC-specific and closely related (papillary thyroid carcinoma) literature discussed above. Two patterns emerge clearly from this synthesis. First, diagnostic performance consistently improves when functional modalities are combined rather than used in isolation, with the best-performing combination in the available literature—SWE plus CEUS—reaching 91% sensitivity and 77% specificity for inconclusive cervical lymph nodes [21]. Second, HNSCC-specific cohorts remain small relative to the broader cervical lymph node literature, with sample sizes ranging from a 13-patient pilot protocol [20] to a 235-patient morphology-focused dataset [8], underscoring the need for larger, prospective, multiparametric-specific HNSCC cohorts called for in Section 6.

## 5. Clinical Applicability and Practical Considerations

The preceding sections established both the biological rationale and the imaging evidence for multiparametric ultrasound as an angiogenesis-informed diagnostic tool. Translating that evidence into routine head and neck practice depends on a separate set of considerations: where ultrasound fits relative to cross-sectional imaging, what it realistically adds at each stage of the patient journey, what it costs the health system to deploy imaging repeatedly, and what practical and organizational barriers currently limit its use.

### 5.1. Comparative Diagnostic Positioning and the Economic Case for Repeatability

Direct head-to-head evidence now exists to situate ultrasound among the available cross-sectional alternatives rather than assuming its role by default. A systematic review and meta-analysis comparing CT, MRI, PET-CT, and ultrasound for detecting cervical lymph node metastasis in clinically node-negative (cN0) HNSCC found that no single modality dominated across sensitivity, specificity, and diagnostic odds ratio: ultrasound performed competitively overall, while PET-CT’s higher sensitivity came at the cost of reduced specificity, largely reflecting inflammatory false positives [28]. This absence of a single superior modality is itself the argument for a complementary rather than hierarchical role for ultrasound within the diagnostic pathway.

That complementary role gains further weight from the economics of head and neck cancer surveillance. A single-institution economic evaluation of 136 patients followed for a median of 3.2 years after treatment found a mean of 14 imaging studies per patient, at a mean total cost of $36,800, with disease-free patients alone averaging $9600 per year in imaging costs—much of it exceeding what National Comprehensive Cancer Network guidelines actually recommend [29]. In a landscape where post-treatment imaging is already demonstrably overused and costly, a radiation-free, low-cost, repeatable modality capable of resolving specific equivocal findings—rather than replacing the surveillance schedule outright—represents a rational way to add diagnostic information without compounding an already documented cost burden.

### 5.2. Biopsy Technique Considerations

Where ultrasound findings prompt histological confirmation, the choice of biopsy technique itself is a live methodological question relevant to how multiparametric findings would be acted upon in practice. A randomized controlled trial comparing ultrasound-guided core needle biopsy (with hydrodissection to protect adjacent critical structures) against conventional fine-needle aspiration for high-risk cervical lymph nodes found the core-needle approach feasible and safe, with diagnostic performance at least matching FNA cytology while providing a larger tissue sample suitable for more extensive histological and immunohistochemical work-up [30]. This matters directly for the argument advanced in this review: if a future validated protocol were to target biopsy toward the specific, angiogenically active region of a node identified by SWE/CEUS co-localization, a core-needle sample from that targeted region would also permit direct histological correlation with angiogenesis markers such as microvessel density—the validation step identified as the central evidence gap in Section 3—in a way that cytology alone cannot.

In current, non-investigational practice, multiparametric findings already inform ultrasound-guided sampling in three concrete ways, none of which require the validated angiogenic-scoring framework proposed in Section 5.6 to be useful today. First, when several cervical nodes are enlarged, Doppler and B-mode features help select which node to sample, prioritizing the one with the most suspicious morphological and vascular profile rather than the most accessible or largest one. Second, within a single node, multiparametric findings help select where to sample: the pilot data discussed in Section 4.2, showing elastographic hardening and irregular enhancement localized to the nodal center rather than a softer, better-perfused cortex [20], argue for directing the needle toward the stiffer, more heterogeneously enhancing region rather than sampling blindly, and the cystic/necrotic considerations discussed in Section 5.7 argue equally for actively avoiding the fluid-predominant center of an HPV-associated node, where a sample is more likely to be non-diagnostic. Third, Doppler flow mapping immediately before or during the procedure serves a practical safety role independent of any diagnostic interpretation, allowing the needle path to be planned around vascular structures. Which technique to use once a target is selected remains guided by what the sample needs to answer: fine-needle aspiration remains sufficient where a cytological diagnosis of malignancy is the immediate clinical question—while core-needle sampling, shown to be feasible and safe for high-risk cervical nodes [30]—is the more appropriate choice whenever architectural or immunohistochemical information is needed, as it already is in current practice for indeterminate nodes where cytology alone has proven insufficient.

### 5.3. Standardized Reporting and the Anticipated Role of Ultrasound

Post-treatment surveillance imaging in head and neck cancer has moved decisively toward structured, standardized reporting. The Neck Imaging Reporting and Data System (NI-RADS), developed by the American College of Radiology, provides a four-tier, risk-stratified lexicon—originally validated for CT and FDG-PET/CT and extended to MRI in a 2025 update—that links standardized descriptions of post-treatment findings directly to management recommendations, reducing inter-radiologist variability that has historically complicated interpretation of the treated neck [31]. A comprehensive 2026 review of the framework explicitly identifies future incorporation of ultrasound, alongside advanced imaging techniques and circulating biomarkers, as a stated direction for the system’s continued evolution [31]—a meaningful signal that the standardization community already anticipates a structured role for ultrasound-derived parameters, rather than treating ultrasound as an uncoordinated adjunct outside the reporting framework altogether.

### 5.4. Surveillance of the Irradiated Neck

Radiotherapy and chemoradiation induce fibrosis and architectural distortion that closely mimic the morphological appearance of persistent or recurrent disease, and this measurably shifts the performance of ultrasound-based assessment. In a comparative series of 111 ultrasound-guided FNA procedures in oral squamous cell carcinoma, sensitivity was 88.0% and specificity 91.4% in patients without prior radiotherapy, versus 97.1% sensitivity and 83.3% specificity in irradiated necks—a pattern consistent with radiotherapy-induced tissue change increasing false-positive morphological suspicion [32]. Building on this, a retrospective analysis of 104 irradiated oral cancer patients with post-treatment lymphadenopathy found that adding ultrasound—using a dedicated post-radiotherapy predictive scoring model—or ultrasound-guided FNA to standard MRI/CT surveillance raised the area under the ROC curve from 0.828 (MRI/CT alone) to 0.920 (with the ultrasound model) and to 0.983 (with ultrasound-guided FNA added) [33]. Neither study incorporated SWE or CEUS, leaving open—and, in the framework of this review, a natural next step—whether functional parameters could further sharpen this already-demonstrated benefit in exactly the setting where morphology is least reliable.

### 5.5. Operator Dependency and the Evidence for Structured Training

Ultrasound’s central practical limitation, reiterated throughout this review, is dependence on operator skill—and this is now supported by direct experimental evidence rather than assumption. A randomized cross-over trial found that a single 6-h formal head and neck ultrasound course measurably improved otolaryngology residents’ diagnostic accuracy from 62% to 75%, with sensitivity rising from 64% to 79% and specificity from 54% to 62%; notably, even residents with substantial prior scanning experience (a mean of 57 prior neck ultrasound examinations) still improved significantly, and the authors concluded that residents had not yet reached a score sufficient for independent practice even after training, underscoring how much structured instruction—rather than passive accumulated exposure—matters [34]. This finding is reinforced by a validated competency-assessment instrument specifically developed for surgeon-performed head and neck ultrasonography, which provides objective, structured criteria for determining when an operator has reached an acceptable proficiency threshold [35]. Together, these data indicate that the operator-dependency limitation affecting every modality discussed in Section 4 is addressable through deliberate, structured training programs—a solvable organizational problem rather than an inherent property of the technology—but also that such training requires formal curricular investment rather than informal on-the-job exposure alone.

### 5.6. Toward Integrated, Evidence-Based Adoption

Taken together, the evidence in this section supports a specific, bounded role for multiparametric ultrasound: a repeatable, cost-conscious complement to cross-sectional imaging [28,29], deployed at defined decision points—equivocal nodal triage, biopsy planning [30], and surveillance of the irradiated neck [32,33]—within an evolving standardized reporting environment that already anticipates its inclusion [31], provided by operators who have completed structured, competency-validated training [34,35]. What remains untested is whether multiparametric ultrasound specifically, layered onto this already-supportive practical infrastructure, delivers measurable improvements in outcomes that matter—appropriateness of neck dissection, time to recurrence detection, or survival—a gap that the Discussion addresses directly.

**Figure 3:**
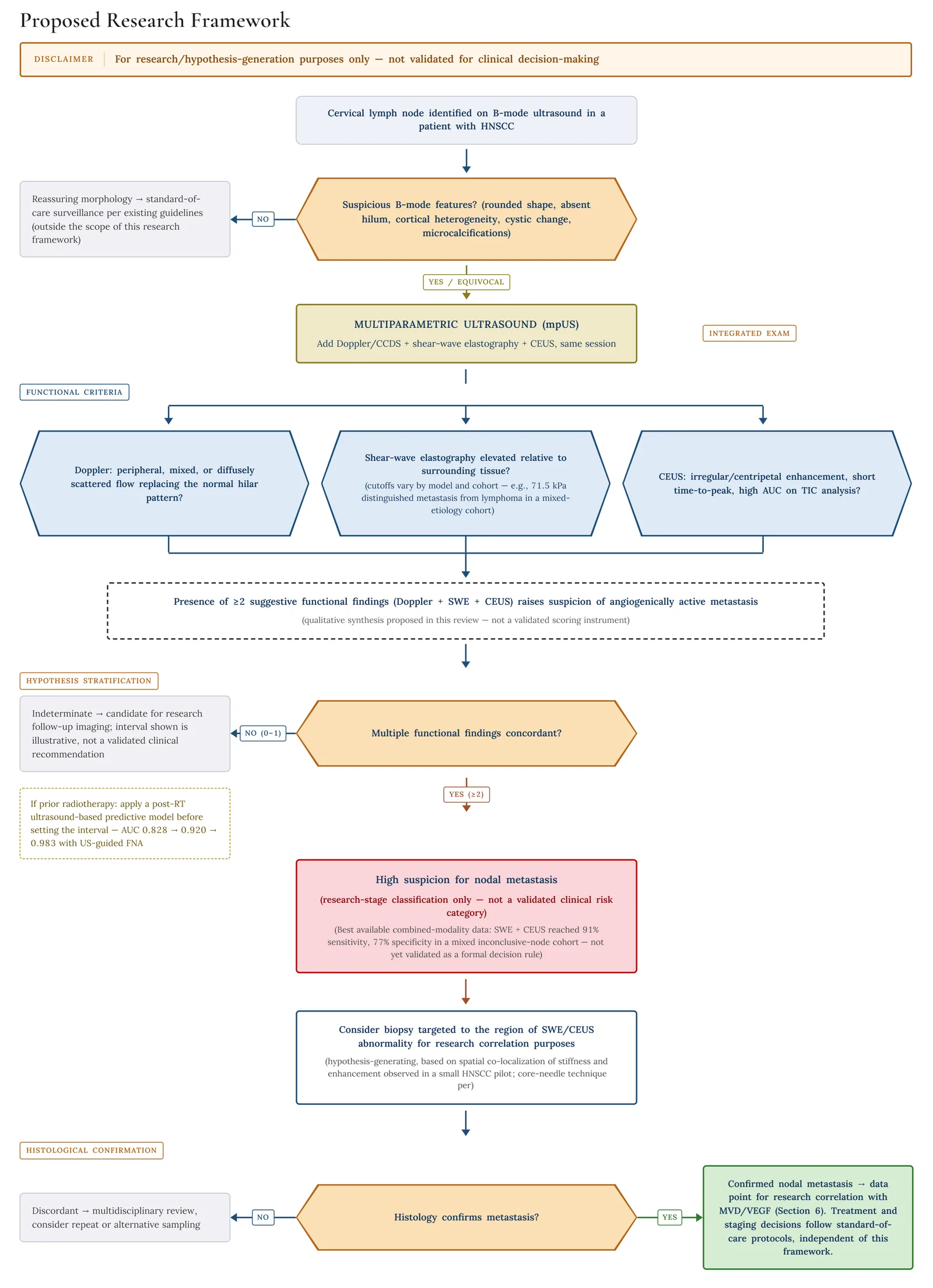
Proposed research framework for integrating multiparametric ultrasound (mpUS) into the study of a cervical lymph node in a patient with head and neck squamous cell carcinoma (HNSCC). In this hypothesis-generating framework, nodes with suspicious or equivocal B-mode features would undergo a combined Doppler/color-coded duplex sonography (CCDS), shear-wave elastography (SWE), and contrast-enhanced ultrasound (CEUS) examination in the same research session. Concordance of multiple functional findings is hypothesized to reflect angiogenically active metastasis, a hypothesis that could be tested by directing research biopsy sampling toward the region of SWE/CEUS abnormality and correlating findings with microvessel density (MVD) and vascular endothelial growth factor (VEGF) expression, as discussed in Section 6. This framework is proposed by the authors as a synthesis of the evidence reviewed in Section 4 and Section 5 for research purposes only; it has not been clinically validated, is not a decision rule for biopsy or treatment planning, and should not currently guide individual patient management. Numerical thresholds and performance figures shown are drawn from the individual studies cited at each step, not from a unified validation cohort. Legend: mpUS, multiparametric ultrasound; CCDS, color-coded duplex sonography; SWE, shear-wave elastography; CEUS, contrast-enhanced ultrasound; TIC, time–intensity curve; AUC, area under the curve; FNA, fine-needle aspiration; RT, radiotherapy; MVD, microvessel density; VEGF, vascular endothelial growth factor [16,20,21,30,33].

Taken together, the considerations reviewed in this section can be synthesized into a proposed research framework for integrating multiparametric ultrasound into the assessment of a cervical lymph node in HNSCC, from initial B-mode triage through functional characterization to histological correlation (Figure 3). This framework is offered as a structured synthesis of the evidence discussed in Section 4 and Section 5, intended to organize future research questions and generate testable hypotheses—not as a validated diagnostic algorithm or a decision rule for biopsy or treatment planning. The thresholds, functional criteria, and decision points it integrates come from separate studies with different cohorts, reference standards, and—in the case of shear-wave elastography cutoffs—different comparator pathologies; the framework as a whole has not been prospectively tested and should not, at the current level of evidence, guide individual patient management.

### 5.7. HPV-Associated Oropharyngeal Carcinoma and Cystic Nodal Metastasis

An increasing proportion of HNSCC, particularly oropharyngeal carcinoma, is driven by high-risk human papillomavirus (HPV), and this subgroup has ultrasound implications that the multiparametric framework discussed above does not yet address. Cystic or necrotic cervical lymph node metastasis is strongly associated with HPV-related disease: in a retrospective series of neck dissections, HPV DNA was identified in 87% of cystic nodal metastases versus none of the solid metastases examined (*p* < 0.0001), and cystic nodes were predominantly linked to a tonsillar primary [36]. This has direct consequences for the multiparametric approach argued for in this review. First, a predominantly cystic node can appear anechoic and well-circumscribed on B-mode; an appearance that overlaps with benign entities such as a second branchial cleft cyst—creating a risk of false reassurance rather than triggering the functional work-up proposed in Section 5.6. Second, Doppler and CEUS interrogation of a cystic or necrotic node preferentially samples the fluid component, which is intrinsically avascular, so vascularity and perfusion parameters measured there may understate angiogenic activity that is confined to a thin, malignant peripheral rim rather than reflect its absence. Third, shear-wave elastography stiffness values in a fluid-containing node are of uncertain reliability and have not been validated against solid nodal cutoffs, so the SWE thresholds discussed in Section 4.2 should not be assumed to transfer to this subgroup. None of the HNSCC-specific multiparametric ultrasound studies reviewed in Section 4 explicitly stratified results by HPV or p16 status, and this represents a specific, addressable gap for future prospective cohorts, alongside the broader validation priorities outlined in Section 6.

## 6. Discussion

This review has argued a specific, three-part thesis: that angiogenesis is a well-characterized biological driver of HNSCC behavior; that multiparametric ultrasound—Doppler, elastography, and CEUS—captures physically continuous correlates of that biology; and that this convergence has a plausible, bounded role in clinical practice at defined decision points. Read together, Section 3, Section 4 and Section 5 make that case coherently. What they do not yet do—and this is the central limitation of the current evidence, not merely of this review’s synthesis of it—is close the loop with direct histological confirmation that multiparametric ultrasound parameters actually track angiogenic activity, as opposed to tracking morphological and structural correlates of malignancy that happen to co-occur with angiogenesis. The clearest existing precedent for that direct correlation comes largely from the adjacent solid-tumor literature that this review’s search deliberately extended into, precisely because HNSCC-specific data are so scarce. In a preclinical xenograft model of nasopharyngeal carcinoma—itself a HNSCC subsite—CEUS perfusion parameters correlated significantly with histologically quantified microvessel density (r up to 0.90) and, more modestly, with VEGF immunohistochemical expression (r = 0.46) [37].

More persuasively, because it is a human, prospective, biopsy-confirmed study rather than an animal model, a cohort of 87 patients with non-small cell lung cancer—34 of them squamous cell carcinoma—found that CEUS time–intensity curve parameters and qualitative “microflow enhancement” patterns correlated significantly with CD34-assessed microvessel density on matched needle-biopsy specimens, with the correlation reported as strongest specifically in the squamous cell carcinoma subgroup [38]. A comparable human correlation has been demonstrated outside the head and neck in breast cancer, where CEUS peak-intensity and ascending-slope ratios corresponded significantly with the peripheral-to-central microvessel density ratio on matched core-needle biopsies [39].

Together, these three studies—one preclinical and HNSCC-adjacent, two human and in other squamous or epithelial tumor types—establish that the underlying premise of this review (CEUS parameters can track histological angiogenesis) is biologically sound and methodologically achievable. What has not yet been published, to the best of this search, is the equivalent prospective, human, HNSCC-specific study correlating multiparametric ultrasound parameters directly against microvessel density and VEGF expression on matched surgical or biopsy specimens. Replicating the Chen et al. [38] or Li et al. [39] designs in cervical lymph nodes or primary HNSCC tumors, rather than lung or breast tissue, is the single most direct and achievable next study this review can point to.

It is also worth situating ultrasound briefly within the broader landscape of angiogenesis-directed functional and molecular imaging in HNSCC, rather than presenting it as the only such approach under investigation. Integrin-targeted PET tracers (RGD-based ligands) have been piloted in HNSCC and shown to visualize a distinct, angiogenesis-specific signal compared with conventional FDG-PET [40], while serial functional MRI/PET during chemoradiation has shown that early changes in perfusion-related parameters predict treatment response before conventional size-based criteria become interpretable—direct HNSCC evidence for the same early-response-monitoring rationale this review proposes for ultrasound in Section 5.3 [41,42]. None of these modalities supersede the others—RGD-PET offers molecular specificity, MRI offers superior soft-tissue contrast, ultrasound offers cost and bedside repeatability—but the parallel is instructive: the reproducibility and validation gaps facing multiparametric ultrasound recur across every functional imaging modality attempting to characterize HNSCC angiogenic biology, including techniques with far greater installed infrastructure and funding than head and neck ultrasound currently receives.

That standardization challenge is not hypothetical. A multi-vendor Quantitative Imaging Biomarker Alliance phantom study directly comparing commercial shear-wave elastography platforms found shear wave speed measurement bias of ±9.6% across elastic phantoms and ±15.3% across viscoelastic phantoms—a magnitude of inter-system variability large enough to plausibly shift a stiffness measurement across a clinically meaningful diagnostic cutoff [43]. This is concrete, quantitative evidence for why the EFSUMB acquisition guidelines discussed in Section 4 and Section 5 are a practical necessity rather than a bureaucratic formality, and it should temper any assumption that a stiffness or perfusion cutoff derived on one ultrasound platform will transfer unchanged to another. The likeliest near-term path toward closing the operator-dependency and quantification gaps identified in Section 5 is computational rather than purely procedural.

Ultrasound-based radiomics and machine learning are already demonstrably feasible in HNSCC cervical lymph node assessment: a model combining texture-analysis radiomics with neural-network classification, trained on 537 lymph nodes from 126 HNSCC patients, achieved an area under the curve of 0.98 for metastasis prediction [44], and quantitative ultrasound radiomics—extracting mid-band fit, spectral slope, and related backscatter parameters rather than conventional B-mode texture features—has been shown capable of predicting post-radiotherapy recurrence in node-positive HNSCC patients [45]. Neither of these radiomics approaches was designed specifically to extract angiogenesis-linked vascular or perfusion features, and neither has yet been validated against the multiparametric (Doppler/SWE/CEUS) protocols discussed in Section 4; integrating radiomic feature extraction directly with the functional, angiogenesis-linked parameters this review has focused on is a natural, currently unrealized next step that would combine the reproducibility benefits of automated quantification with the specific biological rationale this review has argued for.

Several limitations are present in this review and should be stated plainly. It is a narrative rather than a systematic review, and while search terms and inclusion considerations were specified in Section 2, no formal risk-of-bias scoring or PRISMA-style flow diagram was applied. A meaningful share of the strongest quantitative CEUS and elastography evidence for cervical lymph node characterization originates from papillary thyroid carcinoma (PTC) rather than HNSCC directly. We acknowledge this is a substantive limitation, not merely a technical one: PTC and HNSCC differ substantially in angiogenic biology and metastatic nodal phenotype, and extrapolating diagnostic thresholds or biological interpretation from one to the other is not directly justified. These PTC-derived data are retained in this review only for what they can support methodologically—namely, that a given ultrasound technique (e.g., SWE, CEUS) is technically capable of detecting a stiffness or perfusion gradient between benign and malignant cervical nodal tissue in general—and are explicitly flagged at each point of use so the reader can discount them accordingly; they are not used to support any HNSCC-specific angiogenic conclusion, nor should the specific sensitivity and specificity figures quoted in Section 4 be assumed to transfer unchanged to HNSCC biology. HNSCC-specific multiparametric pilot cohorts remain small (13–126 patients across the studies reviewed here) and predominantly single-center, raising the standard concerns about generalizability, operator-specific results, and unquantified publication bias toward positive findings. Framing a research agenda from these gaps is not this review’s invention: it maps directly onto the internationally recognized imaging biomarker development framework proposed by O’Connor et al., which specifies that new imaging biomarkers must cross parallel “translational gaps” of technical (assay) validation, biological/clinical validation, and cost-effectiveness assessment before qualifying for clinical decision-making, rather than being adopted on diagnostic accuracy alone [46]. Applied to multiparametric ultrasound in HNSCC, the technical validation tier is reasonably advanced via the EFSUMB guidelines discussed in Section 4, though the QIBA phantom data show real residual inter-system variability even there [43]; the biological/clinical validation tier—direct histological correlation with MVD and VEGF—remains the binding constraint, achieved elsewhere [37,38,39] but not yet in HNSCC; and the cost-effectiveness tier has barely been addressed beyond the general surveillance-cost context established in Section 5 [29]. Positioned against this formal framework, four concrete priorities follow: (i) prospective, adequately powered HNSCC-specific cohorts directly correlating multiparametric ultrasound parameters against histological microvessel density and VEGF expression on matched specimens, following the design precedent set outside HNSCC [37,38,39]; (ii) standardized, multicenter acquisition protocols enabling pooled analysis across the fragmented single-center literature described throughout Section 4, informed by the measurement-bias data already available for elastography [43]; (iii) integration of automated radiomic and deep-learning quantification specifically with functional (vascular/stiffness) rather than purely morphological ultrasound parameters, building on demonstrated feasibility in HNSCC lymph node classification [44,45]; and (iv) outcome-linked, early-response-monitoring trials analogous to the FDG-PET/functional MRI precedent already established in HNSCC [41], testing whether ultrasound-derived functional change during treatment predicts outcome as reliably as its MRI and PET counterparts, assessed within the biological/clinical and cost-effectiveness validation tiers specified by the imaging biomarker roadmap [46]. Until this agenda is substantially advanced, multiparametric ultrasound should be regarded as a biologically well-motivated and practically promising imaging biomarker of angiogenic activity, not yet an established non-invasive surrogate for angiogenesis, and as a complement to existing head and neck imaging facing the same validation challenges as its MRI and PET counterparts [40,41,42] rather than lagging uniquely behind them.

## 7. Conclusions

Multiparametric ultrasound: combining B-mode, Doppler, shear-wave elastography, and contrast-enhanced ultrasound offers a radiation-free, low-cost, repeatable window onto the vascular phenotype of HNSCC that is biologically continuous with tumor angiogenesis. Current evidence, drawn from a narrative rather than systematic search and still limited to small, largely single-center, and in part HNSCC-adjacent (papillary thyroid carcinoma) cohorts without formal risk-of-bias assessment, indicates that combining these modalities can improve discrimination of malignant cervical lymph nodes beyond conventional morphology alone, with plausible clinical value in biopsy guidance, pre-treatment baseline characterization, and surveillance of the irradiated neck. This heterogeneity and the acknowledged methodological limitations of the underlying literature, detailed in Section 6, mean that the relationship between angiogenesis and multiparametric ultrasound findings should be regarded as biologically plausible and inferential rather than firmly established. In particular, no study has yet directly correlated multiparametric ultrasound parameters with histological microvessel density and VEGF expression in HNSCC patients, the key step separating a promising imaging biomarker from a validated angiogenic surrogate. Prospective, multicenter, histologically anchored studies addressing this gap are needed before multiparametric ultrasound can be recommended for routine use in this indication.

## Figures and Tables

**Table 1:** Summary of key studies on multiparametric ultrasound of cervical lymph nodes in head and neck squamous cell carcinoma and related models.

Study	Year	Design (n)	Modality	Key Finding
Sproll et al. [8]	2023	Prospective; 235 patients/4539 LNs (HNSCC)	B-mode	Subjective B-mode sensitivity 79%, specificity 99% for nodal metastasis
de Koekkoek-Doll et al. [23]	2021	Prospective; 102 patients/211 nodes (HNSCC)	B-mode + MFI + RI	Peripheral vascularity on micro-flow imaging predicts malignancy
Jiang et al. [9]	2019	Retrospective; PTC cohort	CEUS + SWE	Combined CEUS + elastography model outperforms either modality alone
Künzel et al. [20]	2022	Prospective pilot; 13 patients (HNSCC)	B-mode + SWE + CEUS	Elastographic hardening co-localizes with irregular CEUS enhancement in the same nodal core
Termure et al. [16]	2025	Prospective; 41 malignant LNs (mixed etiology, mostly OSCC)	SWE	Benign < 35 kPa; metastasis ≥ 50 kPa; final model AUC 0.837
Spiesecke et al. [26]	2022	Meta-analysis; 5 studies (cervical LN)	CEUS + B-mode	Sensitivity 76% → 92%, specificity 80% → 91% (B-mode alone vs. combined with CEUS)
Wakonig et al. [21]	2023	Prospective; 105 patients/107 nodes (inconclusive CLN)	Full mpUS (B-mode + CCDS + SWE + CEUS)	SWE + CEUS combination: 91% sensitivity, 77% specificity
Suh et al. [25]	2016	Meta-analysis; 8 studies, 481 patients	SWE	Pooled sensitivity 81%, specificity 85% for malignant cervical LN

LN, lymph node(s); HNSCC, head and neck squamous cell carcinoma; PTC, papillary thyroid carcinoma; OSCC, oral squamous cell carcinoma; MFI, micro-flow imaging; RI, resistive index; SWE, shear-wave elastography; CEUS, contrast-enhanced ultrasound; CCDS, color-coded duplex sonography; AUC, area under the curve.

## Data Availability

No new data were created or analyzed in this study. Data sharing is not applicable to this article.

## Abbreviations

The following abbreviations are used in this manuscript:

AUC	Area under the curve
cN0	Clinically node-negative
CCDS	Color-coded duplex sonography
CEUS	Contrast-enhanced ultrasound
CLN	Cervical lymph node(s)
CT	Computed tomography
DCE	Dynamic contrast-enhanced
EFSUMB	European Federation of Societies for Ultrasound in Medicine and Biology
FNA	Fine-needle aspiration
HIF-1α	Hypoxia-inducible factor 1-alpha
HNSCC	Head and neck squamous cell carcinoma
kPa	Kilopascal
MFI	Micro-flow imaging
mpUS	Multiparametric ultrasound
MRI	Magnetic resonance imaging
MVD	Microvessel density
NI-RADS	Neck Imaging Reporting and Data System
OSCC	Oral squamous cell carcinoma
PET	Positron emission tomography
PTC	Papillary thyroid carcinoma
QIBA	Quantitative Imaging Biomarker Alliance
RGD	Arginine-glycine-aspartate
RI	Resistive index
ROC	Receiver operating characteristic
RT	Radiotherapy
SWE	Shear-wave elastography
TIC	Time–intensity curve
TNM	Tumor, node, metastasis
VEGF	Vascular endothelial growth factor
VEGFR	Vascular endothelial growth factor receptor
